# Accurate identification of abnormal ploidy using an artificial intelligence model in preimplantation genetic testing

**DOI:** 10.1093/hropen/hoaf054

**Published:** 2025-09-02

**Authors:** Pingyuan Xie, Rijing Pang, Luyao Zeng, Shuoping Zhang, Lei Sun, Kaisen Yang, Xiaoyi Yang, Shuang Zhou, Senlin Zhang, Guangjian Liu, Yueqiu Tan, Liang Hu, Fei Gong, Jia Fei, Ge Lin

**Affiliations:** Hunan Guangxiu Hospital, Hunan Normal University Health Science Center, Changsha, China; Clinical Research Center for Reproduction and Genetics in Hunan Province, Reproductive and Genetic Hospital of CITIC-Xiangya, Changsha, China; National Engineering and Research Center of Human Stem Cells, Changsha, China; Hunan Guangxiu Hospital, Hunan Normal University Health Science Center, Changsha, China; Hunan Guangxiu Hospital, Hunan Normal University Health Science Center, Changsha, China; Clinical Research Center for Reproduction and Genetics in Hunan Province, Reproductive and Genetic Hospital of CITIC-Xiangya, Changsha, China; Peking Jabrehoo Med Tech Co. Ltd, Beijing, China; Hunan Guangxiu Hospital, Hunan Normal University Health Science Center, Changsha, China; Clinical Research Center for Reproduction and Genetics in Hunan Province, Reproductive and Genetic Hospital of CITIC-Xiangya, Changsha, China; National Engineering and Research Center of Human Stem Cells, Changsha, China; Hunan Guangxiu Hospital, Hunan Normal University Health Science Center, Changsha, China; Peking Jabrehoo Med Tech Co. Ltd, Beijing, China; Clinical Research Center for Reproduction and Genetics in Hunan Province, Reproductive and Genetic Hospital of CITIC-Xiangya, Changsha, China; NHC Key Laboratory of Human Stem Cell and Reproductive Engineering, Institute of Reproductive and Stem Cell Engineering, School of Basic Medical Science, Central South University, Changsha, China; Clinical Research Center for Reproduction and Genetics in Hunan Province, Reproductive and Genetic Hospital of CITIC-Xiangya, Changsha, China; NHC Key Laboratory of Human Stem Cell and Reproductive Engineering, Institute of Reproductive and Stem Cell Engineering, School of Basic Medical Science, Central South University, Changsha, China; Clinical Research Center for Reproduction and Genetics in Hunan Province, Reproductive and Genetic Hospital of CITIC-Xiangya, Changsha, China; NHC Key Laboratory of Human Stem Cell and Reproductive Engineering, Institute of Reproductive and Stem Cell Engineering, School of Basic Medical Science, Central South University, Changsha, China; Peking Jabrehoo Med Tech Co. Ltd, Beijing, China; Clinical Research Center for Reproduction and Genetics in Hunan Province, Reproductive and Genetic Hospital of CITIC-Xiangya, Changsha, China; National Engineering and Research Center of Human Stem Cells, Changsha, China; NHC Key Laboratory of Human Stem Cell and Reproductive Engineering, Institute of Reproductive and Stem Cell Engineering, School of Basic Medical Science, Central South University, Changsha, China

**Keywords:** triploidy, single pronucleus-derived embryos, tripronucleate embryo, preimplantation genetic testing, genome-wide uniparental diploidy, artificial intelligence

## Abstract

**STUDY QUESTION:**

Can ultra-low-coverage whole-genome sequencing (ulc-WGS) accurately identify abnormal ploidy during preimplantation genetic testing (PGT)?

**SUMMARY ANSWER:**

The artificial intelligence (AI)-based PGT-Plus model demonstrates high accuracy in ploidy detection, offering a cost-effective solution that enhances clinical utility of PGT.

**WHAT IS KNOWN ALREADY:**

The predominant PGT for aneuploidy can identify chromosomal aneuploidies but cannot determine ploidy status. Transferring embryos with ploidy abnormalities can result in miscarriage and molar pregnancy. On the other hand, in ART, fertilization is assessed by morphological pronuclear assessment at the zygote stage. However, it has a low specificity in the prediction of abnormal ploidy status and embryos deemed abnormally fertilized can yield healthy pregnancies. Accurately identified abnormal ploidy in PGT-A can resolve current limitations and expand the utility range of PGT-A. Several studies have identified ploidy abnormalities; however, they were mainly based on single-nucleotide polymorphism (SNP) arrays or needed to combine additional targeted-next-generation sequencing (NGS) information. Studies based on ulc-WGS remain scarce.

**STUDY DESIGN, SIZE, DURATION:**

The study consisted of two stages: methodology establishment and validation. An AI model, named PGT-Plus, was developed using 653 samples with known ploidy status, which was further validated using 792 different ploidy status samples. In the clinical application stage, the approach was used to analyse the ploidy status of 19 103 normally fertilized PGT blastocysts and 140 single pronucleus (1PN)-derived blastocysts collected between May 2022 and December 2023. All blastocysts were tested using trophectoderm biopsy and NGS.

**PARTICIPANTS/MATERIALS, SETTING, METHODS:**

The methodology is based on the ulc-WGS data. First, based on samples with known ploidy status: the heterozygosity rate of high-frequency biallelic SNPs, the likelihood ratio (LLR) of alleles was calculated under different assumptions (‘both parental homologs’ [BPH] from a single parent, ‘single parental homolog’ [SPH] from each parent, disomy, and monosomy) by leveraging allele frequencies and linkage disequilibrium (LD) measured in the 1000 genomes project database. Twenty-three continuous candidate features derived from heterozygosity rates and LLRs of chromosomes or selected windows were included to establish the ploidy prediction AI model. Gini importance analysis and multicollinearity mitigation was performed for feature selection, then the performance of Random Forest (RF), Support Vector Machine (SVM), and Logistic Regression for modelling was compared. Subsequently, the parameter optimization was performed based on the RF model. Ploidy constitution concordance was evaluated in known ploidy status samples. The frequency of abnormal ploidy in normal fertilized PGT blastocysts and 1PN-derived blastocysts (including conventional IVF and ICSI) was evaluated.

**MAIN RESULTS AND THE ROLE OF CHANCE:**

Eleven features were collected for model architecture compared to SVM and Logistic Regression; RF achieved superior performance for ploidy detection. The AI model achieved an AUC of 1 for genome-wide-uniparental diploidy (GW-UPD), 1 for triploidy, and 0.99 for diploidy. For the 792 validation samples, 99.5% of samples were successfully detected using the AI model, and the model showed 100% accuracy for ploidy classification. In the clinical application stage, out of 19 103 PGT samples, 19 069 were successfully analysed using the model, with 110 (0.57%) identified as having abnormal ploidy embryos. Among these, 12.7% (14/110) were identified as GW-UPD, and 87.3% (96/110) were triploid. Among 5563 diploid blastocysts transferred, 3478 clinical pregnancies were achieved. Subsequent ploidy analysis was performed for 217 spontaneous abortion and 935 prenatal diagnostic samples, and no abnormal ploidy was identified. Furthermore, of the 140 1PN embryos tested, 40 (28.6%) exhibited GW-UPD, 3 (2.1%) exhibited triploidy, and 97 (69.3%) were determined to be biparental and normally fertilized. Among the 97 biparental embryos, 46 were diploid, 11 were mosaic, and 40 were aneuploid. In terms of the insemination pattern, the percentage of abnormal ploidy in ICSI was significantly higher than in conventional IVF (*P* < 0.01, 37.1% vs. 2.9%, respectively). With full informed consent, 20 patients without euploidy from normal fertilization chose 1PN-derived biparental and diploid blastocysts to transfer, resulting in 10 clinical pregnancies and 9 ongoing pregnancies.

**LARGE-SCALE DATA:**

N/A.

**LIMITATIONS, REASONS FOR CAUTION:**

Some rare ploidy abnormalities, such as polyploidy with an equal number of identical sets of chromosomes and ploidy mosaicism cannot be accurately identified. Moreover, the origin of abnormal ploidy was not identified due to the unavailability of DNA from both parents.

**WIDER IMPLICATIONS OF THE FINDINGS:**

The PGT-Plus AI model provides a ploidy evaluation method based on the conventional PGT-A data and integrates directly into standard PGT-A workflows. Clinical utility results suggest that the model is a valuable tool for identifying embryos with abnormal ploidy in PGT-A and rescuing normal diploid embryos from abnormally fertilized embryos. These findings demonstrate that PGT-Plus significantly enhances the diagnostic accuracy of PGT.

**STUDY FUNDING/COMPETING INTEREST(S):**

This study was supported by grants from Major Scientific Program of CITIC Group (No. 2023ZXKYB34100, to Ge.L.), Hunan Provincial Grant for Innovative Province Construction (2019SK4012), Hunan Xiangjiang New District (Changsha High-tech Zone) key core technology research project in 2023, and Science Foundation of Hunan Province (Grant 2023JJ30422). All authors declared no conflicts of interest..

WHAT DOES THIS MEAN FOR PATIENTS? Human reproduction is not highly efficient, and chromosomal abnormalities in embryos significantly contribute to implantation failure and pregnancy loss.

Preimplantation genetic testing for aneuploidy (PGT-A) is a genetic testing method commonly used in IVF to avoid transfers of aneuploid embryos (embryos with an abnormal number of chromosomes). Our artificial intelligence (AI)-based model, PGT-Plus provides a more comprehensive genetic analysis of embryos, detecting complex abnormalities that standard PGT might overlook—such as triploidy (the presence of an additional set of chromosomes). This advanced screening helps prevent the transfer of unsuitable embryos (which occurs in about 0.57% of cases), reducing the risk of pregnancy complications. In addition, women with advanced age or low ovarian reserve often have few usable embryos. Some embryos labelled ‘abnormally fertilized’ during early checks were previously discarded. PGT-Plus can now identify chromosomally normal embryos from these embryos (with a 30–40% normal rate), increasing pregnancy opportunities. In conclusion, the PGT-Plus automated testing process does not require extra embryo handling and helps doctors and patients make better-informed decisions.

## Introduction

Human reproduction is not highly efficient, and chromosomal abnormalities significantly contribute to implantation failure and pregnancy loss in embryos, whether through natural conception or IVF (Capalbo *et al.*, 2017a; Essers *et al.*, 2023). To improve outcomes, preimplantation genetic testing for aneuploidy (PGT-A) is commonly used in IVF by avoiding aneuploid embryo transfers (Tan *et al.*, 2014; Treff and Zimmerman, 2017; Ma *et al.*, 2023). Currently, the predominant clinical PGT-A methodologies are next-generation sequencing (NGS)-based platforms that rely only on whole-genome amplification and comparative chromosome copy number analysis, which can accurately identify chromosomes and segment aneuploidies (Xie *et al.*, 2022a). However, due to low coverage, these methods cannot detect genome-wide chromosomal anomalies, such as triploidy, genome-wide uniparental diploidy (GW-UPD), and haploidy, which can lead to molar pregnancies, embryonic lethality, and developmental disorders (Staessen and Van Steirteghem, 1997; McFadden and Robinson, 2006; Xu *et al.*, 2016).

Triploidy is the presence of an additional set of chromosomes in the cell, resulting in a total of 69 chromosomes. The extra-haploid set can be of maternal or paternal origin and is referred to as digynic or diandric triploidy, respectively (McFadden and Robinson, 2006). As a common chromosomal anomaly, triploidy occurs in approximately 1–3% of all conceptuses and contributes to 8–10% of all spontaneous abortions (McFadden and Robinson, 2006; Essers *et al.*, 2023). Uniparental diploidy (UPD) occurs when both copies of a chromosome are inherited from the same parent, with the other parent’s chromosome being absent (Engel, 1980). This is termed GW-UPD when it refers to an entire chromosome set. GW-UPD has severe developmental consequences; for example, paternal GW-UPD is lethal *in utero* as it manifests as a hydatidiform mole, occurring at a rate of 0.05–1.3% of pregnancies (Bo *et al.*, 2024).

In ART, fertilization is assessed by counting the pronuclear bodies 16–18 hours after insemination. Normal fertilization is confirmed by the presence of two pronuclear (2PN) bodies and two polar bodies. During IVF/ICSI cycles, the frequencies of single pronucleus (1PN) and tri-pronuclear (3PN) derived embryos are approximately 1–8% and 1–7% (Balakier *et al.*, 1993; Capalbo *et al.*, 2017a; Xie *et al.*, 2018), respectively. However, morphological assessment of PN does not always correlate with ploidy status. For example, not all zygotes with two pronuclei are diploid; approximately 0.5–1% abnormal ploidy was observed in blastocysts biopsied for PGT-A (Marin *et al.*, 2018; Caroselli *et al.*, 2023; Kratka *et al.*, 2023). Additionally, embryos derived from zero-pronuclear oocytes (0PN), 1PN, and 3PN do not always exhibit abnormal ploidy. Previous investigations have revealed that approximately 50% of 1PN and 10% 3PN-derived embryos are diploid (Destouni *et al.*, 2018; Xie *et al.*, 2018; Alteri *et al.*, 2024). Thus, it is essential to identify embryos with abnormal ploidy using the present NGS-based PGT platform.

Given the widespread application of NGS-based PGT, several studies have attempted to establish a platform for identifying ploidy aberrations using NGS methods (Xie *et al.*, 2022a; Caroselli *et al.*, 2023), such as targeted NGS, linkage-based genotyping, and haplotype analysis. However, these approaches often require additional procedural steps and extra cost, limiting their extensive clinical application. To address these limitations, we developed PGT-Plus, an artificial intelligence (AI) model designed to predict ploidy classification based on routine PGT-A low-coverage genomic data. This approach eliminates the need for additional experimental procedures while maintaining diagnostic accuracy, offering a cost-effective and streamlined solution for widespread clinical applications. After confirming the accuracy of the method, it was used to evaluate the ploidy abnormalities of 19 103 2PN embryos derived from 7038 PGT cycles and 140 blastocysts derived from 1PN embryos.

## Materials and methods

### Ethics statement

This study was approved by the Ethics Committee of CITIC-Xiangya Reproductive & Genetic Hospital (LLSC-2023–026). Written informed consent was obtained from all participants.

### Study design

**Figure 1.**
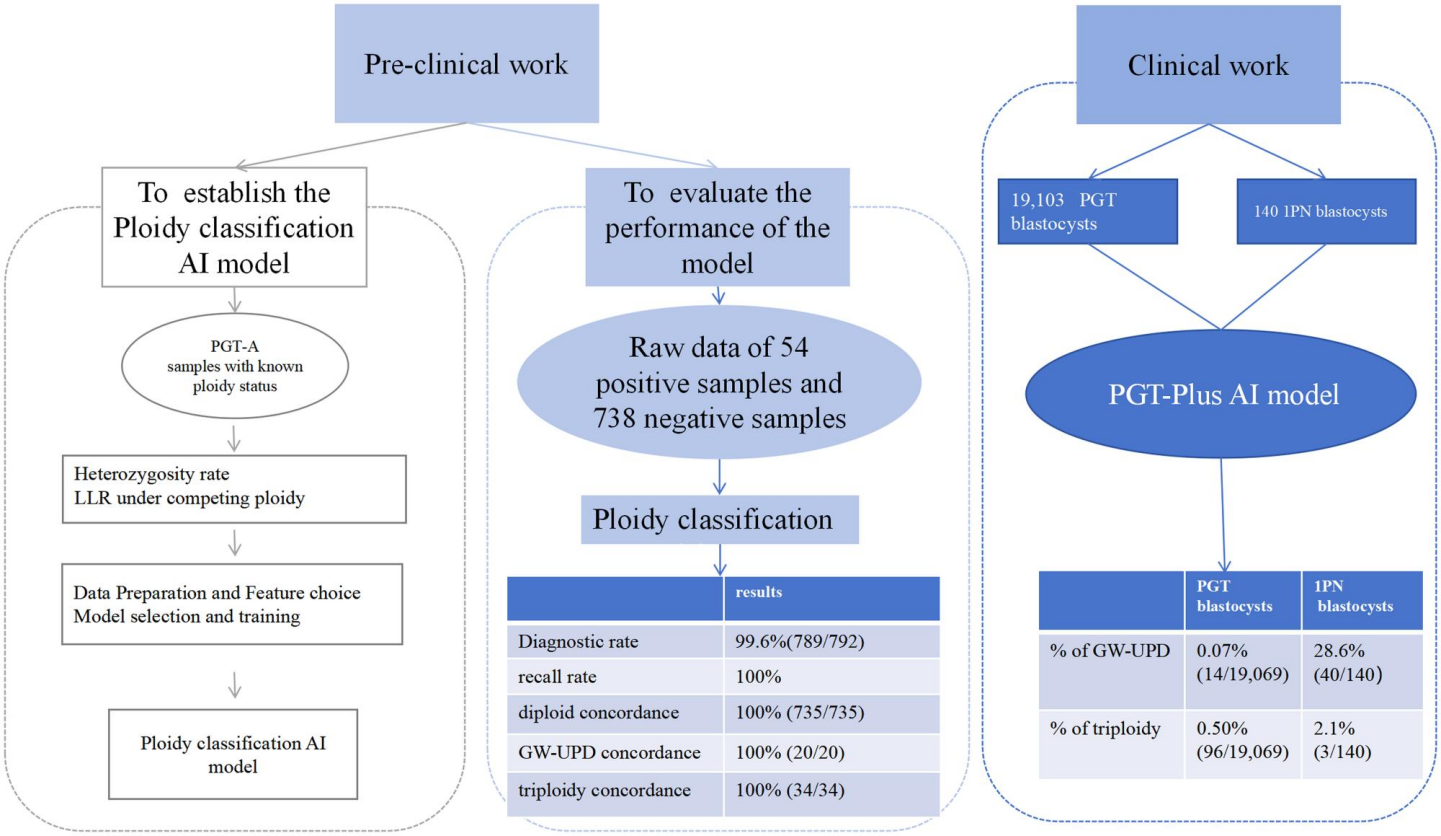
**Study workflow overview.** This study comprises of two phases: The left panel illustrates the preclinical work, including the establishment of the Ploidy classification AI model and its performance evaluation. The right panel shows the clinical application of the PGT-A Plus model, encompassing 19 243 PGT blastocysts. PGT-A: preimplantation genetic testing for aneuploidy; AI: artificial intelligence; PGT: preimplantation genetic testing; LLR: likelihood ratio; GW-UPD: genome-wide-uniparental diploidy; 1PN: single pronucleus.

An integrated bioinformatics pipeline was developed based on an existing PGT-A sequencing workflow. The pipeline is capable of analysing copy number variations (CNVs) greater than 1 Mb and 30–70% mosaicism more than 10 Mb. It also assesses UPD and triploidy simultaneously. Two stages were included: (i) in preclinical testing, we first developed an AI model using 653 known samples (188 normal diploids, 126 heterozygous triploids, 20 samples with whole-genome regions of homozygosity [ROH], 271 simulated ROH samples derived from consanguineous pedigrees/segmental deletion/monosomy samples, and 48 simulated contaminated samples using data admixture with different samples), all previously characterized by single-nucleotide polymorphism (SNP)-array analysis. The ploidy prediction AI model was validated using 792 samples with known ploidy status, which included 738 diploids corresponding to transferred embryos resulting in healthy newborns, 34 triploids, and 20 UPD samples, all previously characterized by SNP-array analysis or Haplo-PGT analysis (Xie *et al.*, 2022b). (ii) In the clinical application stage, 19 243 biopsy samples (19 103 2PN samples from 7038 PGT-A/SR cycles and 140 samples from 1PN embryos) were tested using the PGT-Plus AI model to analyse copy number variation and ploidy (Fig. 1).

### Sample preparation

All samples including 653 samples for model development, 792 samples for validation, and 19 243 samples for clinical application were provided by the Reproductive and Genetic Hospital of CITIC-Xiangya between May 2022 and December 2023. The study materials were acquired after obtaining written informed consent from all donor couples, ensuring ethical compliance and respect for the donors’ willingness to contribute to this research.

Samples for clinical application were collected from 7038 PGT cycle. Of the 7038 PGT-A/SR cycles, 5704 were PGT-A cycles (clinical indications included advanced maternal age ≥38 years, recurrent implantation failure [RIF] with ≥2 failures, recurrent pregnancy loss [RPL] with ≥2 miscarriages, and previous aneuploid conception), while 1334 were PGT-SR cycles (clinical indications included reciprocal translocation, Robertsonian translocations, insertional translocations, deletions, duplications, and inversions).

In addition, 140 1PN-derived blastocysts were collected for analysis. At our centre, patients with 1PN embryos (including both conventional IVF insemination and ICSI) undergo comprehensive genetic counselling to evaluate the clinical necessity of PGT-Plus for selecting normal diploid blastocysts. Typically, patients with advanced maternal age or diminished ovarian reserve, who exhibit limited embryo availability, opted for ploidy detecting to optimize transfer outcomes.

### Ovarian stimulation, fertilization assessment, embryo culture, and blastocyst biopsy and transfer

PGT procedures were conducted at the Reproductive and Genetic Hospital of CITIC-Xiangya as previously described (Xie *et al.*, 2022a; Zhang *et al.*, 2023). Ovarian stimulation was performed based on the patient’s status. ICSI was used for all metaphase II oocytes. Normal fertilization was assessed 16–18 hours after ICSI by visualizing two pronuclei and two polar bodies. 1PN embryos were identified when the number of pronuclei was 1. All embryos were cultured in sequential media (G1 and G2; Vitrolife, Gothenburg, Sweden) to the blastocyst stage under 6% carbon dioxide, 5% oxygen, and 89% nitrogen in a MINC Mini incubator (Cook Medical, Bloomington, IN, USA) or an APM-50D incubator (Astec Industries, Chattanooga, TN, USA). On days 5, 6, and 7, blastocysts were evaluated using the Gardner grading system (Capalbo *et al.*, 2014). A trophectoderm (TE) biopsy was performed on days 5, 6, or 7, and approximately 3–5 cells were collected after zona pellucida dissection and laser-assisted hatching (ZILOS-tk, Hamilton Thorne, Beverly, MA, USA). Blastocysts were cryopreserved as previously described. Biopsied TE cells were washed three times in G-MOPS medium and then either used directly for whole genome amplification (WGA) or stored at −80°C for future WGA. Only normal diploid/mosaic blastocysts that survived and re-expanded were considered for transfer.

Embryonic trophoblast samples were processed using the PicoPLEX WGA kit (Rubicon Genomics, Ann Arbor, MI, USA) for PGT-A and the REPLI-g Single-Cell Kit (QIAGEN, Germany) for PGT-SR using multiple displacement amplification (MDA)-based WGA, according to the manufacturers' instructions. Sequencing was performed on the Illumina NextSeq 550 platform (Illumina, San Diego, CA, USA) or the MGI-200 platform (BGI, Shenzhen, China). After removing adapters and low-quality sequences, high-quality reads were aligned to the human hg19 genome using BWA software (Li and Durbin, 2009). Picard (https://broadinstitute.github.io/picard/) was used to remove duplicate sequences, and only uniquely mapped reads were used for subsequent CNV calculations. Reads were divided into continuous 600Kb windows based on their genomic mapping positions, with read counts as the coverage of each window. Data normalization was performed using classical GC, mappability, and baseline calibrations. Calibrated window coverage was used for CNV boundary detection using the HMM (hidden Markov model) algorithm. CNV boundaries were used to segment the genome into fragments, with the median calibrated coverage of all windows in each fragment representing the copy ratio of the fragment. Ultimately, the state of each fragment was determined based on the copy ratio: a copy ratio above 23 indicated chromosome/fragment duplication, with a ratio between 23 and 27 indicating mosaic duplication. A copy ratio below 17 indicated chromosome/fragment deletion, with a ratio between 13 and 17 indicating mosaic deletions.

### Data preparation and feature choice

High-frequency biallelic SNPs (minor allele frequency: 0.01–0.5) from the dbSNP database were used as target sites. The sequencing depths of these sites were determined and filtered to calculate the autosomal heterozygosity rate for each sample. A Z-score for heterozygosity rate was calculated using baseline data means and standard deviation (SD). ROH were identified via a 1 Mb sliding window approach, with the heterozygosity rate calculated for each window. Consecutive windows exhibiting heterozygosity rates below a threshold of 0.02 were merged to define ROH boundaries and their corresponding heterozygosity values were collected. The Z-score and Euclidean distance between the ROH and the normal chromosomal regions were also calculated. Chromosomes were divided into 100 kb windows, and haplotype frequencies were determined for reads covering sites in the haplotype database (1000 genomes project) for each window. The likelihood ratio (LLR) of alleles on sequencing reads was then calculated under different assumptions (‘both parental homologs’ [BPH] from a single parent, ‘single parental homolog’ [SPH] from each parent, disomy, and monosomy) (Ariad *et al.*, 2021). Finally, the distribution of the LLRs was evaluated through multiple sampling, and 100 kb windows were merged into larger segments. From heterozygosity rates and LLR-derived metrics (e.g. ratios, proportions, counts, means, Z-score, SD), a total of 23 candidate features were initially collected. All 23 candidate features were continuous variables (Supplementary Table S1). Initially, 23 candidate features derived from heterozygosity rates and LLRs were considered. After Gini importance analysis, 12 features with the highest discriminative power were selected for the final model. Further, to address the multicollinearity problem among features, a systematic processing pipeline was performed. First, highly correlated feature pairs were identified using Pearson correlation coefficient matrices (threshold |r| > 0.8) and partitioned into interconnected feature groups via connected components analysis. Subsequently, variance inflation factor (VIF) analysis was integrated with feature importance ranking to perform selective filtering. Within each feature group, only the highest-importance feature was retained while others were eliminated. Concurrently, standalone features not belonging to any group were removed if they exhibited both VIF > 10 and importance below the global median (0.033). The optimized feature set was reduced to 11 (Supplementary Table S2); mean VIF decreased from 679.5 to 155.6. Model accuracy increased by 2.4% to 0.8929. GW-ROH was identified based on the heterozygosity rates.

### Model selection and training

To determine the optimal model for ploidy prediction, we first performed an initial evaluation of three algorithms—RF, SVM, and Logistic Regression—using 23 features with 378 (from 653 known samples cohort) known samples, and compared the AUC, precision, recall, and F1-score. Subsequently, the parameters of the RF model were initialized, including setting the number of decision trees to 100, adopting a cross-validation strategy, independently training each tree on different subsamples of the dataset, and evaluating the model performance over 10 iterations for performance optimization. Then, in the parameter optimization phase, the maximum depth of each tree was set to 15 to prevent the model from becoming overly complex, thereby reducing the risk of overfitting. In addition, the minimum sample number required for internal node repartitioning and the minimum sample number for leaf nodes were set to further enhance the general stability of the model. A comprehensive ensemble of trees was constructed by repeating the steps above. Predictions were then made using a majority-vote mechanism, and their predictive performance was evaluated using receiver operating characteristic (ROC) curves. Based on the model’s performance on the validation set, parameters such as the number of decision trees and the maximum number of features per tree were further adjusted to improve the model’s ability to predict diploid, triploid, and ROH events. Finally, the optimized and evaluated RF model was applied to real-world prediction tasks of diploid, triploid, and ROH events.

### Statistical analysis

The predictive accuracy of models was assessed using ROC curve analysis, with the AUC as a quantitative measure of performance. Statistical analyses were performed using SPSS (v18.0, IBM, Armonk, NY, USA). The chi-square (χ^2^) test was used to compare frequencies between different insemination pattern groups, embryo quality groups, and biopsy time groups. A *P*-value of <0.05 was considered statistically significant.

## Results

### Ploidy probability prediction using an AI model

**Figure 2.**
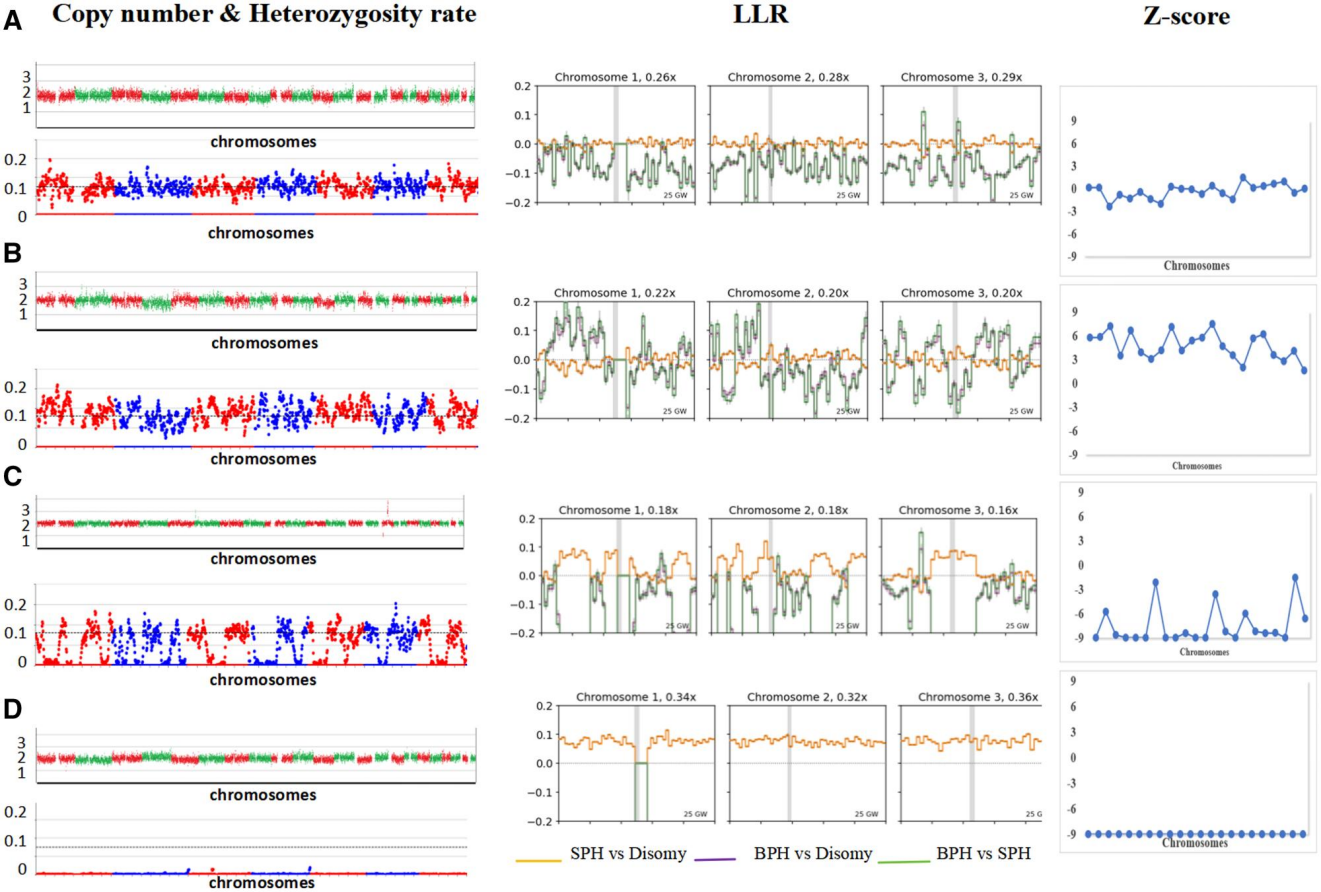
**Diploidy, triploidy, and genome-wide-uniparental diploidy exhibited characteristic heterozygosity rate plots, likelihood ratio plots, and Z-score.** Copy number results showed no difference in different samples. For diploidy, the LLR between disomy and BPH or SPH both indicated disomy in all chromosomes (**A**). For triploidy (**B**), the LLR between disomy and BPH indicated BPH in most chromosomes. Due to homologous recombination, crossed large successive BPH regions were seen in most chromosomes. Hybrid GW-UPD showed large ROH in each chromosome, and the LLR comparison between disomy and SPH indicated SPH (**C**). For GW-UPD, homozygous GW-UPD exhibited loss of heterozygosity globally, and the LLR comparison between disomy and monosomy indicated monosomy (**D**). The absolute value of Z-score for heterozygosity rate of each chromosome was very high in triploidy and GW-UPD. GW-UPD: genome-wide-uniparental diploidy; BPH: both parental homologs; SPH: single parental homolog; ROH: regions of homozygosity; LLR: likelihood ratio.

Genotyping signatures of triploidy, GW-UPD, and haploidy were distinguished from normal diploidy (Supplementary Fig. S1). We first integrated the heterozygosity rate value and LLR under competing ploidy hypotheses (BPH, SPH, disomy, and monosomy). Results from 653 samples with known ploidy statuses were analysed. Homozygous GW-UPD embryos exhibited global loss of heterozygosity, and the LLR comparison between disomy and monosomy indicated monosomy. Hybrid GW-UPD embryos showed large ROH in each chromosome, with LLR comparisons between disomy and SPH indicating SPH (Fig. 2). In triploidy, due to homologous recombination, the LLR comparison between disomy and SPH manifests as switches between the BPH and SPH tracts. The LLR comparisons between disomy and BPH indicated BPH on several chromosomes (usually more than 10) (Fig. 2).

To establish an automated and precise methodology for Ploidy prediction, we tried to develop an AI model. First, 23 candidate features derived from heterozygosity rates and LLRs of chromosomes or selected windows were included in the analysis. Then the performance of RF, SVM, and Logistic Regression was compared; RF achieved superior performance with mean AUC = 1.00 for triploidy detection, compared to SVM (AUC = 0.99) and logistic regression (AUC = 0.98) and the precision, recall, F1-score. (Supplementary Table S3 and Supplementary Fig. S2).

**Figure 3.**
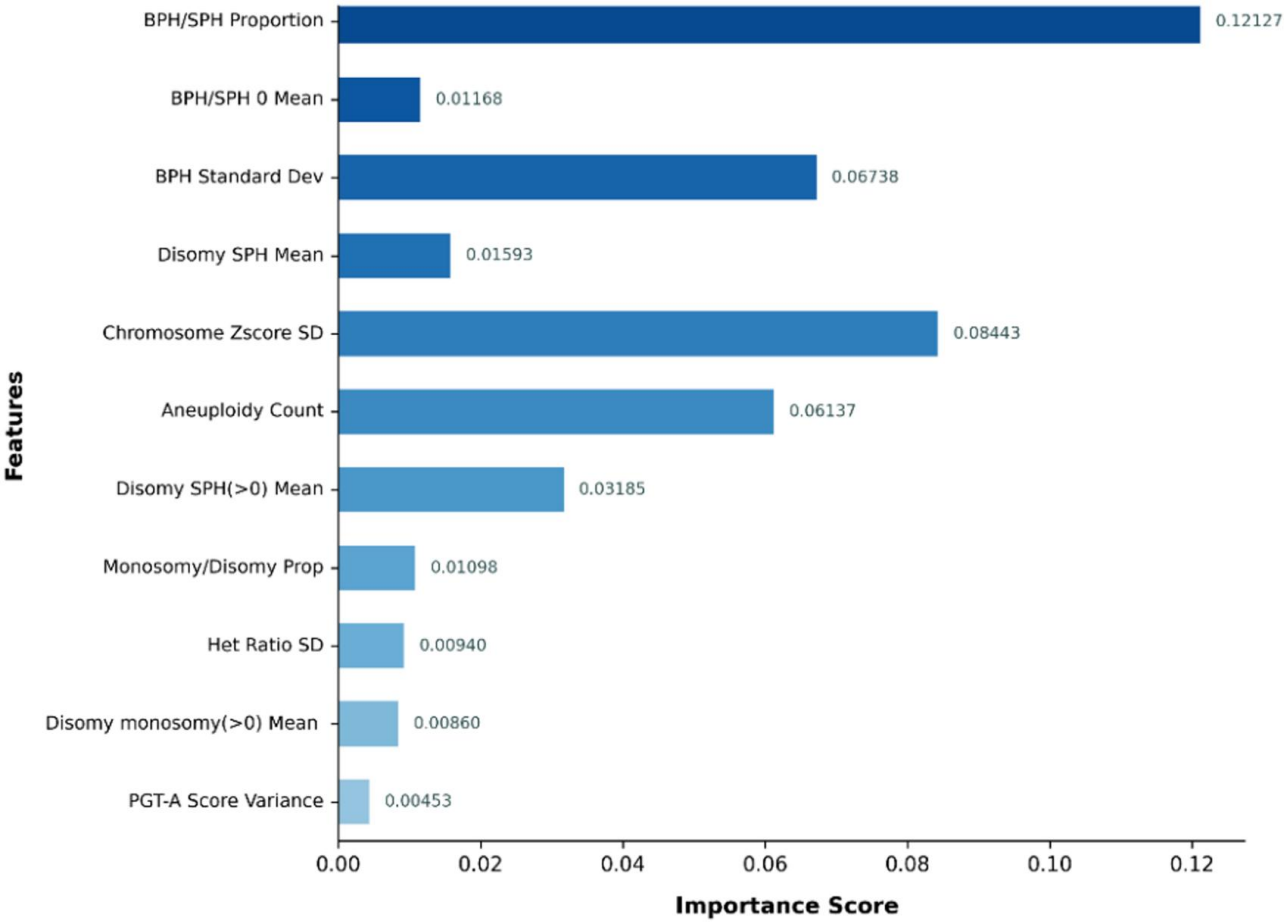
**Feature importance scores of various factors in the RF model for ploidy prediction.** GW-UPD: genome-wide-uniparental diploidy; BPH: both parental homologs; SPH: single parental homolog; ROH: regions of homozygosity; LLR: likelihood ratio; SD: standard deviation; PGT-A: preimplantation genetic testing for aneuploidy; RF: Random Forest; BPH/SPH Proportion: the proportion of autosomal windows with LLRs (BPH_vs_SPH) >0 relative to total windows.; BPH/SPH 0 Mean: mean LLR (BPH_vs_SPH) of autosomal windows with LLR >0.; BPH Standard De: SD of LLRs for BPH_vs_disomy across 22 chromosomes.; Disomy SPH Mean: mean LLR (SPH_vs_disomy) across all autosomal windows.; Chromosome Zscore SD: SD of z-scores across all autosomes.; Aneuploidy Count: number of euploid abnormalities in PGT-A results.; Disomy SPH(>0) Mean: mean LLR (SPH_vs_disomy) of autosomal windows with LLR >0.; Monosomy/Disomy Prop: the proportion of windows (monosomy_vs_disomy) exhibiting LLRs greater than zero relative to the total window count on autosomal.; Het Ratio SD: SD of heterozygosity ratios per chromosome across all autosomes.; Disomy monosomy(>0) Mean: mean LLR (monosomy_vs_disomy) of autosomal windows with LLR >0.; PGT-A Score Variance: SD of PGT-A results.

**Figure 4.**
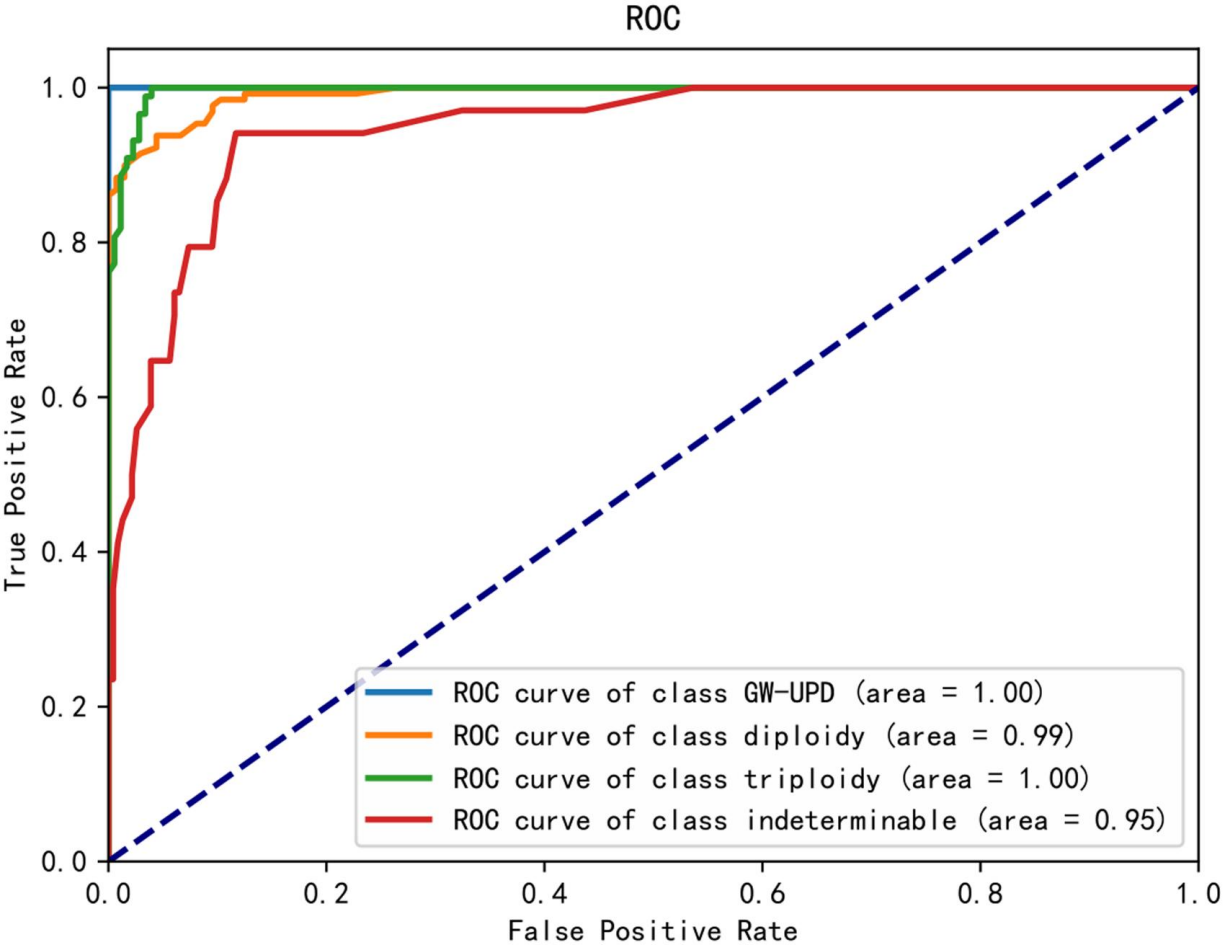
**ROC curves of PGT-Plus model for classifying diploidy, triploidy, GW-UPD and indeterminable.** ROC: receiver operating characteristic; PGT: preimplantation genetic testing; GW-UPD: genome-wide-uniparental diploidy.

Based on the RF model, Gini importance analysis was employed to assess the features’ importance. Based on the understanding of feature significance and the proportion of Gini importance, we removed relatively unimportant features. Other variables with VIF ≥10 were excluded to mitigate multicollinearity. Eleven features were ultimately collected for integration into the final model architecture (Fig. 3). Further, the parameter optimization was performed based on validated dates. The AI model for ploidy classification demonstrated an AUC of 0.95–1 and an accuracy of 0.9–1, depending on the sample type (Fig. 4).

Finally, 54 positive and 738 negative samples were collected, and a blinded analysis was performed using the AI model (PGT-Plus). Among 738 diploid samples, corresponding to embryos that were successfully transferred and resulted in healthy newborns, ploidy analysis was informative in 735 samples, with a detection success rate of 99.6%. All 735 diploid samples, as well as 54 triploid and GW-UPD samples, were 100% concordant with the expected results. This demonstrates that PGT-Plus can accurately detect triploidy and GW-UPD with 100% recall rate sensitivity and specificity.

### Triploid and GW-UPD embryos in 2PN PGT blastocysts

Based on the excellent performance of the PGT-Plus validation results, the analytical strategy was first used for 19 103 PGT-A/SR 2PN samples derived from 7038 PGT cycles in 5300 patients (including 5704 PGT-A cycles and 1334 PGT-SR cycles). In this cohort, the ploidy results of 34 samples were indeterminable, 110 (0.57%) were identified as abnormally ploidy embryos. Among the 110 embryos, 96 blastocysts from 94 patients were categorized as triploid, resulting in a triploidy frequency of 0.50% (96/19069) in human 2PN blastocysts. Of the triploid embryos, 44 were identified with an XXX karyotype (27 had additional chromosomal abnormalities), and 52 had an XXY karyotype (24 had additional chromosomal abnormalities). The analysis of the association between embryo quality and triploidy revealed triploidy frequencies of 0% (0/684), 0.15% (4/2660), 0.41% (34/8270), and 0.77% (58/7489) in excellent, good, average, and poor blastocysts, respectively. No significant differences (*P* > 0.05) were observed between maternal age and the incidence of abnormal ploidy (Supplementary Table S4). The prevalence of GW-UPD was 0.07% (14/19 069), including 11 homozygous UPD and 3 hybrid UPD cases. Only one GW-UPD embryo had additional chromosomal abnormalities.

A total of 5563 blastocysts were transferred, resulting in 3478 clinical pregnancies. Among these, 217 samples obtained from 402 spontaneous abortion cases underwent ploidy analysis, all demonstrating diploid chromosomal status, which validates the accuracy of the ploidy prediction model.

### Identification of biparental and diploid blastocysts from 1PN blastocysts

A small subset of 1PN blastocysts are diploid embryos that result from normal fertilization and are valuable for transplantation. We used this analytical strategy to select biparental and diploid blastocysts from 1PN blastocysts. In total, 140 1PN blastocysts from 135 cycles were analysed. Of these, 40 (28.6%) exhibited GW-UPD, 3 (2.1%) exhibited triploidy, and 97 (69.3%) were determined to be biparental and normally fertilized. Among the biparental blastocysts, 46 were identified as euploid, 11 as mosaic, and 40 as aneuploid.

In terms of the insemination pattern, the percentage of abnormal ploidy was significantly higher in embryos produced by ICSI compared to conventional IVF (37.1% vs. 2.9%, respectively, *P* < 0.01; Table 1). In terms of the biopsy time, in the ICSI group, 21.9% of embryos biopsied on days 5 and 6 were GW-UPD embryos. However, 63.4% of embryos biopsied on day 6.5, and day 7 were GW-UPD embryos, with a significant difference (*P* < 0.01, Supplementary Table S5).

**Table 1.** Ploidy results distribution of 1PN blastocysts according to the insemination pattern.

	IVF (n %)	ICSI (n %)	*P*-value
GW-UPD	1 (2.9%)	39 (37.1%)	*P* < 0.01
triploidy	0 (0%)	3 (2.9%)	NS
euploidy	13 (37.1%)	33 (31.4%)	NS
mosaic	5 (14.3%)	6 (5.7%)	NS
aneuploidy	16 (45.7%)	24 (22.9%)	NS
Total	35 (100%)	105 (100%)	

GW-UPD: genome-wide-uniparental diploidy; NS: no significance.

Furthermore, 20 patients chose to transfer the 1PN-derived biparental and diploid blastocysts in the absence of normally fertilized transferable embryos. Among these, 50.0% (10/20) resulted in clinical pregnancies, with nine ongoing pregnancies and six healthy live births (five male and one female neonate).

## Discussion

In this study, we report a novel and cost-effective PGT-Plus AI model for accurately identifying ploidy abnormalities based on a conventional PGT-A platform. After its broad clinical application, favourable clinical outcomes were observed.

Conventional NGS-based PGT platforms are unable to directly distinguish ploidy abnormalities (e.g. triploidy, haploidy, or GW-UPD). This limitation poses two critical clinical challenges: (i) Failure to detect ploidy anomalies increases the likelihood of nonviable embryo transfers and miscarriage. (ii) Embryo wastage: abnormally fertilized embryos based on the morphological pronuclear assessment are routinely discarded. Some approaches have been established to detect both aneuploidies and ploidy abnormalities simultaneously, one is targeted NGS and the other is Linkage-based genotyping analysis and haplotyping. Both can accurately identify ploidy abnormalities. However, targeted NGS requires a specialized targeted panel; linkage-based genotyping analysis is not the current global clinical norm for most IVF cycles and needed maternal and paternal DNA, which would add extra cost. The PGT-Plus AI prediction model integrates a machine learning-driven analytical pipeline into the standard PGT-A workflow. Crucially, this method achieves automated, high-accuracy identification of ploidy abnormalities without requiring additional experimental interventions, parental samples, and the diagnostic performance of this model remains unaffected by AMA (results not shown).

PGT-Plus can accurately identify triploidy and GW-UPD in 2PN-derived PGT blastocysts, thereby avoiding the transfer of blastocysts with abnormal ploidy. Several studies have reported the detection and frequency of abnormal ploidy in blastocysts (Xu *et al.*, 2016; Marin *et al.*, 2018; Walters-Sen *et al.*, 2022; Kratka *et al.*, 2023). In Kratka *et al*.'s study using the SNP array, a 1.43% prevalence of abnormal ploidy was detected, with triploidy (68%) being the most common abnormal ploidy diagnosis (Kratka *et al.*, 2023). Marin *et al.*'s study exhibited a triploidy frequency of 0.474% in 18 791 human blastocysts using a targeted NGS-based comprehensive chromosome screening platform (Marin *et al.*, 2018). SNP-based ploidy analysis detected abnormal results in 1.8% of 128 991 embryos using the FAST-SeqS assay, triploidy being the most common (73.6%) (Walters-Sen *et al.*, 2022). The overall incidence of triploidy is between 0.4% and 1%. We speculate that this fluctuation may be attributed to several factors: (i) insemination method–in conventional IVF, dispermy is a potential cause of triploidy, which can be reduced by ICSI; (ii) the method and stringency of fertilization assessment criteria. For example, the conventional PN assessment was checked at 16–18 hours after insemination, and several studies suggested that time-lapse imaging allows for more timely and accurate fertilization checks (Barrie *et al.*, 2021; Capalbo *et al.*, 2024).

PGT-Plus can accurately identify biparental diploid blastocysts derived from 1PN zygotes. In ART, inspection of pronucleus formation is a routine procedure for selecting 2PN zygotes. According to the guidelines issued by ESHRE in 2016, the transfer of embryos derived from one or zero PNs is not advised even in the absence of available embryos with two PNs for transplantation (De Los *et al.*, 2016). However, several previous studies have shown that a considerable proportion of 1PN-derived embryos are diploid and therefore, suitable for potential clinical use (Capalbo *et al.*, 2017b; Xie *et al.*, 2018; Tong *et al.*, 2023). Capalbo *et al.* suggested most (69.2%) 1PN-derived blastocysts were diploid, and only a few were haploid (23.1%) or triploid (7.7%) (Capalbo *et al.*, 2017b), which is similar to our results.

Our results showed that 37.1% of 1PN blastocysts derived from ICSI were GW-UPD, while IVF-derived 1PN blastocysts had a greater than 97% chance of diploidy. Consistent with this, one study showed the LBR of 1PN blastocysts without ploidy testing was significantly higher in the IVF group than in the ICSI group (33.14% vs. 15.25%) (Li *et al.*, 2020). The precise reasons for the differences between IVF and ICSI remain unclear. In IVF insemination, we speculated that the sperm entered the region close to the MII spindle and had a higher likelihood of fusing with maternal chromosomes, which is normal fertilization. Conversely, in ICSI-derived 1PN embryos, where the sperm is injected from a location far from the spindle, the chances of female and male pronuclei fusion are reduced. Our findings confirm that blastocyst culture alone is insufficient to select normal 1PN embryos. However, GW-UPD 1PN embryos exhibit minor delays in reaching the blastocyst stage and poor embryo quality.1PN blastocysts with good embryo quality and normal developmental stages have a higher likelihood of developing into normal blastocysts. For clinical management of 1PN embryos, we recommend blastocyst culture followed by differential strategies: molecular genetic ploidy assessment may not be routinely required for IVF-derived 1PN blastocysts, particularly those with excellent morphology grading. Conversely, molecular genetic ploidy assessment was suggested for ICSI-derived 1PN blastocysts to exclude ploidy abnormalities before transfer. This may provide an extra opportunity for viable 1PN embryos in ART cycles. Our results indicated that after PGT-Plus, the ongoing pregnancy rate for 1PN blastocyst transfer was 45%, which was significantly higher than that without PGT ploidy testing (21.5–26.3%) (Capalbo *et al.*, 2024). Collectively, the PGT-Plus method was effective for selecting biparental diploid embryos for transfer from 1PN blastocysts, especially ICSI-inseminated embryos.

This study had some limitations: (i) Some ploidy abnormalities cannot be accurately identified, such as (a) polyploidy with an equal number of identical sets of chromosomes, such as 92, XXXX, or 92, XXYY; (b) GW-UPD with two identical chromosomal sets from one parent without recombination; (c) ploidy mosaicism, including mosaic GW-UPD and mosaic triploidy. Fortunately, these cases are extremely rare (Sahoo *et al.*, 2017; Mastromoro *et al.*, 2023). (ii) Contamination remains a potential factor affecting the model’s prediction accuracy due to its complexity. Fortunately, in most cases, contamination from sperm or granulosa cells can be effectively ruled out through standard operation procedures, in additional, utilizing parents genomic DNA, parental contamination can be identified utilizing LLR-based or SNP-based analyses (Bo *et al.*, 2024). (iii) The sample size of 1PN-derived blastocysts remains insufficient for robust statistical analysis. (iv) The observed perfect AUC (1.00) may reflect model overfitting; therefore, systematic evaluation of generalizability through larger, multicentre prospective validation cohorts should be conducted in future studies.

This study successfully developed a PGT-Plus model for predicting chromosomal ploidy abnormalities using low-coverage NGS, which is highly accurate and does not require additional experimental manipulation. The wide clinical application results suggest that this method enhances the clinical utility of PGT by avoiding the transfer of embryos with abnormal ploidy derived from 2PN embryos and rescuing embryos with a normal diploid constitution for clinical use from 1PN blastocysts. Future studies should focus on optimizing AI-driven models to enhance the clinical utility of PGT-A, specifically the ability to distinguish mosaicism and aneuploidy. Furthermore, this approach may elucidate the origin of aneuploidy by distinguishing whether chromosomal abnormalities arise from mitotic errors or meiotic divisions (MII/MI phases).

This study successfully established a PGT-Plus AI model for predicting chromosomal ploidy abnormalities using low-coverage NGS, demonstrating high accuracy without requiring additional experimental manipulation. Clinical validation across 19 103 embryo analyses revealed its dual clinical utility: effectively preventing the transfer of abnormal ploidy embryos derived from 2PN embryos while rescuing 57 viable euploid/mosaic embryos from 140 1PN blastocysts for transfer. Future research should prioritize the optimization of AI models to enhance the diagnostic performance of PGT-A, particularly through the integration of LLR-based methods to distinguish between mitotic- and meiotic-origin chromosomal errors. These advancements would significantly improve the accuracy of detecting both aneuploidies and mosaicism, thereby refining embryo selection strategies in ART.

## Supplementary Material

hoaf054_Supplementary_Data

## Data Availability

The next-generation sequencing datasets supporting this study have not been deposited in public repository because of privacy and ethical restrictions but are available from the corresponding author on request. The PGT-Plus AI model has been integrated into the PGT-A analysis workflow and developed into a web-based application. Due to data security and privacy protection requirements, the platform is not publicly accessible; for access requests, please contact the Corresponding Authors J.F. and G.L.
