# Supplementary material for: Accurate identification of abnormal ploidy using an artificial intelligence model in preimplantation genetic testing
Source: Hum Reprod Open. 2025 Sep 2;2025(4):hoaf054. doi: 10.1093/hropen/hoaf054 (PMC12453672; doi:10.1093/hropen/hoaf054)
Supplement: hoaf054_Supplementary_Data [file hoaf054_supplementary_data.zip › supplementary_figures.pdf]

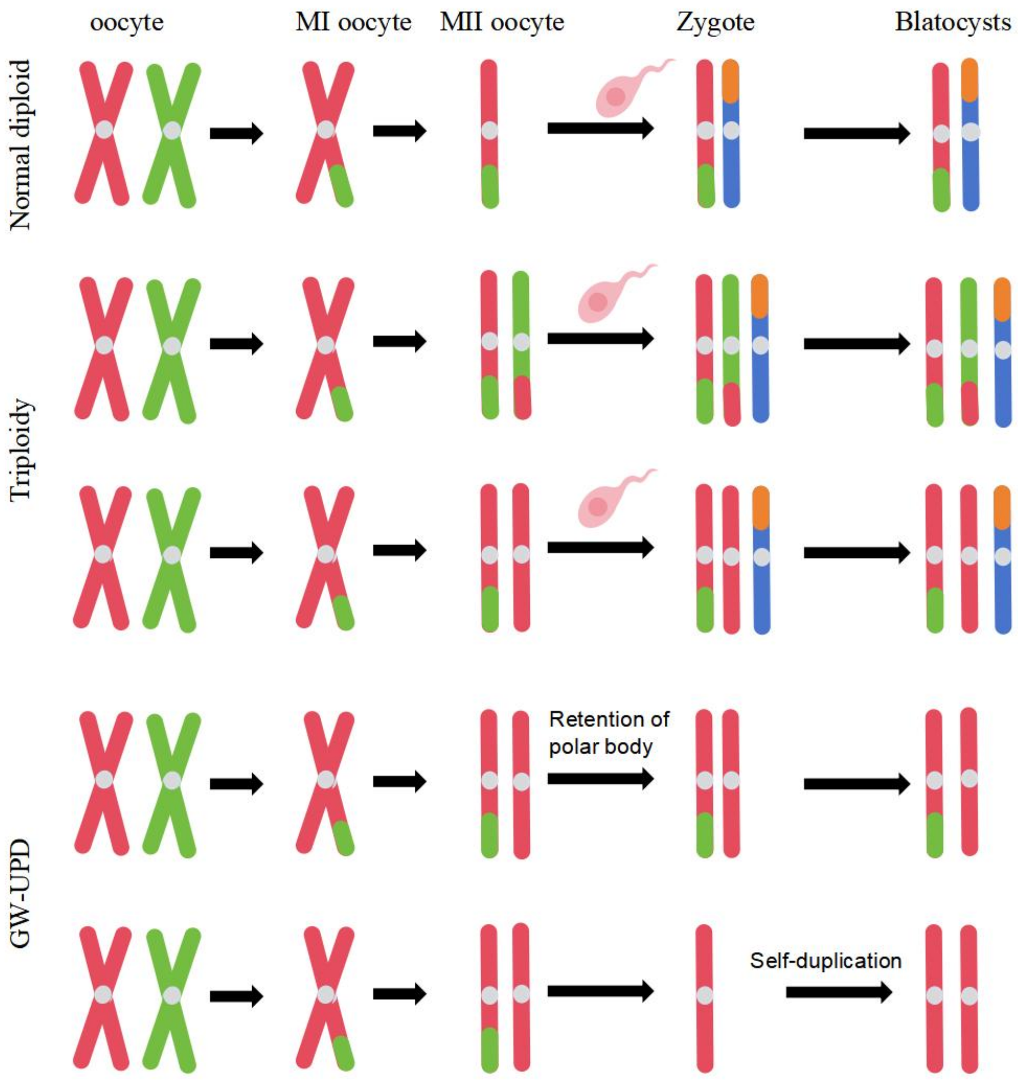

Supplementary Figure S1: Signatures of normal diploidy, triploidy, and GW-UPD with respect to their composition of identical and distinct parental homologs. Normal diploidy has two genetically distinct copies of each chromosome—one copy from each parent—that comprise mosaics of two homologs from each parent. Meiotic-origin triploidy has three distinct parental homologs on a chromosome-wide scale; however, due to homologous recombination, each chromosome has regions with identical and distinct parental homologs, with breakpoints depending on the location of the homologous recombination cross over. GW-UPD features a diploid copy number attributed to only one parent.

GW-UPD: genome-wide-uniparental diploidy

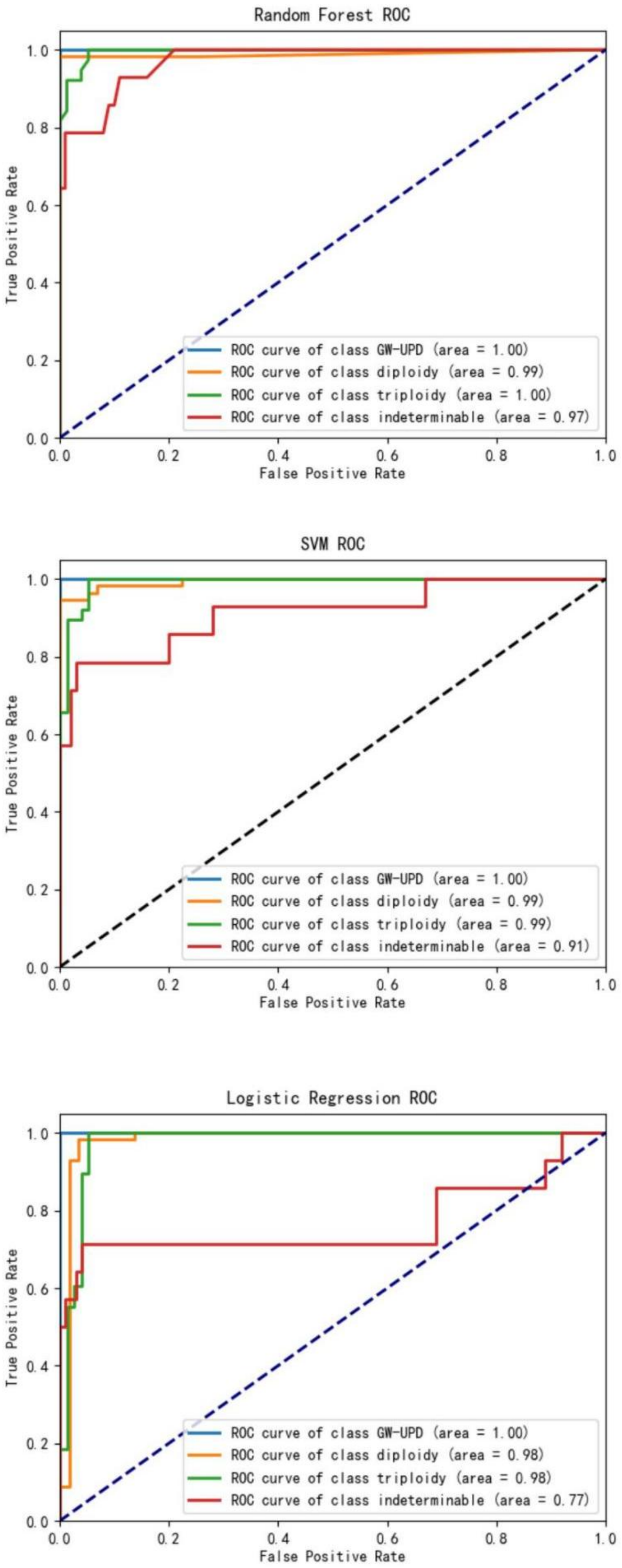

Supplementary Figure S2: ROC curves of Three Models for classifying diploidy, triploidy, GW-UPD and indeterminable.

ROC, receiver operating characteristic；GW-UPD: genome-wide-uniparental diploidy
