# Supplementary material for: Accurate identification of abnormal ploidy using an artificial intelligence model in preimplantation genetic testing
Source: Hum Reprod Open. 2025 Sep 2;2025(4):hoaf054. doi: 10.1093/hropen/hoaf054 (PMC12453672; doi:10.1093/hropen/hoaf054)
Supplement: hoaf054_Supplementary_Data [file hoaf054_supplementary_data.zip › supplementary_tables_1-5_Clean_EO.docx]

Supplementary Table S1 The details of 23 candidate features

|  | charactes | variables type | character importance | Interpretation |
| --- | --- | --- | --- | --- |
|  | Features | variables type | character importance | Interpretation |
| 1 | BPH_num_proportion | continuous | 0.089613 | The proportion of BPH chromosomes among all autosomes under the competing ploidy hypotheses of BPH and disomy. |
| 2 | BPH/disomy_continuity_windows_proportion | continuous | 0.093492 | The proportion of BPH continuity windows (BPH_vs_disomy) relative to the total window count‌. |
| 3 | BPH/disomy_proportion | continuous | 0.078675 | The proportion of windows (BPH_vs_disomy) exhibiting likelihood ratios greater than zero relative to the total window count on autosomal |
| 4 | Z-score | continuous | 0.032647 | Zscore value of heterozygosity rate |
| 5 | BPH/disomy_continuity_overlap_windows_nums | continuous | 0.014747 | The number of continuity overlap (BPH_vs_disomy) windows |
| 6 | BPH/disomy_mean | continuous | 0.089716 | The mean likelihood ratio (BPH_vs_disomy) across all autosomal windows. |
| 7 | BPH/SPH Proportion | continuous | 0.121274 | The proportion of autosomal windows with likelihood ratios (BPH_vs_SPH) >0 relative to total windows. |
| 8 | BPH_SPH_mean | continuous | 0.035821 | Mean likelihood ratio (BPH_vs_SPH) across all autosomal windows. |
| 9 | BPH/SPH 0 Mean | continuous | 0.011683 | Mean LLR (BPH_vs_SPH) of autosomal windows with LLR >0. |
| 10 | BPH Standard De | continuous | 0.067381 | SD of LLRs for BPH_vs_disomy across 22 chromosomes. |
| 11 | monosomy/disomy_mean | continuous | 0.033179 | Mean likelihood ratio (monosomy_vs_disomy) across all autosomal windows. |
| 12 | Disomy SPH Mean | continuous | 0.01593 | Mean LLR (SPH_vs_disomy) across all autosomal windows. |
| 13 | Chromosome Zscore SD | continuous | 0.084432 | SD of z-scores across all autosomes. |
| 14 | Aneuploidy Count | continuous | 0.061374 | Number of euploid abnormalities in PGT-A results. |
| 15 | transitions_num | continuous | 0.043354 | Number of chromosomal recombination events. |
| 16 | chrom_bph_sd | continuous | 0.03783 | Standard deviation (SD) of BPH vs disomy likelihood ratios per chromosome across autosomes. |
| 17 | Disomy SPH(>0) Mean | continuous | 0.031848 | Mean LLR (SPH_vs_disomy) of autosomal windows with LLR >0. |
| 18 | Monosomy/Disomy Prop | continuous | 0.01985 | The proportion of windows (monosomy_vs_disomy) exhibiting LLRs greater than zero relative to the total window count on autosomal |
| 19 | Het Ratio SD | continuous | 0.009403 | SD of heterozygosity ratios per chromosome across all autosomes. |
| 20 | BPH_disomy_0_mean | continuous | 0.008623 | Mean likelihood ratio (BPH_vs_disomy) of autosomal windows with LLR >0. |
| 21 | Disomy monosomy(>0) Mean | continuous | 0.008595 | Mean LLR (monosomy_vs_disomy) of autosomal windows with LLR >0. |
| 22 | SPH/disomy_proportion | continuous | 0.006004 | The proportion of windows (SPH_vs_disomy) exhibiting likelihood ratios greater than zero relative to the total window count on autosomal |

BPH, both parental homologs; SPH, single parental homolog; ROH, regions of homozygosity; LLR, likelihood ratio; PGT-A, preimplantation genetic testing for aneuploidy

Supplementary Table S2 features used in the final triploidy, normal ploidy and indeterminable prediction model

| No | Features | type | importance | Interpretation |
| --- | --- | --- | --- | --- |
| 1 | BPH/SPH Proportion | continuous | 0.121274 | The proportion of autosomal windows with LLRs (BPH_vs_SPH) >0 relative to total windows. |
| 2 | BPH/SPH 0 Mean | continuous | 0.011683 | Mean LLR (BPH_vs_SPH) of autosomal windows with LLR >0. |
| 3 | BPH Standard De | continuous | 0.067381 | SD of LLRs for BPH_vs_disomy across 22 chromosomes. |
| 4 | Disomy SPH Mean | continuous | 0.01593 | Mean LLR (SPH_vs_disomy) across all autosomalwindows. |
| 5 | Chromosome Zscore SD | continuous | 0.084432 | SD of z-scores across all autosomes. |
| 6 | Aneuploidy Count | continuous | 0.061374 | Number of euploid abnormalities in PGT-A results. |
| 7 | Disomy SPH(>0) Mean | continuous | 0.031848 | Mean LLR (SPH_vs_disomy) of autosomal windows with LLR >0. |
| 8 | Monosomy/Disomy Prop | continuous | 0.01985 | The proportion of windows (monosomy_vs_disomy) exhibiting LLRs greater than zero relative to the total window count on autosomal |
| 9 | Het Ratio SD | continuous | 0.009403 | SD of heterozygosity ratios per chromosome across all autosomes. |
| 10 | Disomy monosomy(>0) Mean | continuous | 0.008595 | Mean LLR (monosomy_vs_disomy) of autosomal windows with LLR >0. |
| 11 | PGT-A Score Variance | continuous | 0.004532 | SD of PGT-A results. |

BPH, both parental homologs; SPH, single parental homolog; ROH, regions of homozygosity; LLR, likelihood ratio; PGT-A, preimplantation genetic testing for aneuploidy

Supplementary Table S3 ‌Comparative results of Three Models

|  | Random Forest (RF) | | | Support Vector Machine (SVM) | | | Logistic Regression | | |
| --- | --- | --- | --- | --- | --- | --- | --- | --- | --- |
|  | precision | recall | f1-score | precision | recall | f1-score | precision | recall | f1-score |
| GW-ROH | 1 | 1 | 1 | 1 | 0.833 | 0.909 | 1 | 1 | 1 |
| Diploidy | 0.902 | 0.982 | 0.94 | 0.981 | 0.946 | 0.964 | 0.932 | 0.982 | 0.957 |
| Triploidy | 0.905 | 1 | 0.95 | 0.884 | 1 | 0.938 | 0.884 | 1 | 0.938 |
| Indeterminable | 1 | 0.357 | 0.526 | 0.833 | 0.714 | 0.769 | 1 | 0.429 | 0.6 |

Supplementary Table S4 The frequency of abnormal ploidy according to the maternal age

| age | No of total embryos | No of abnormal ploidy | percentage |
| --- | --- | --- | --- |
| <35 years | 8768 | 48 | 0.5% |
| 35-37 years | 4452 | 24 | 0.5% |
| 38-40 years | 3623 | 20 | 0.6% |
| 41-42 years | 1548 | 7 | 0.5% |
| >42 years | 712 | 11 | 1.5% |

Supplementary Table S5 Ploidy results distribution of 1PN blastocysts according to the biopsy time in ICSI group

|  | ≤144H （n %） | ≥152H （n %） | P-value |
| --- | --- | --- | --- |
| GW-UPD | 14 (21.9%) | 26 (63.4%) | P<0.01 ** |
| triploidy | 2 (3.1%) | 1 (2.4%) | NS |
| euploidy | 26 (40.7%) | 7 (17.1%) | P<0.05 * |
| mosaic | 4 (6.3%) | 2 (4.9%) | NS |
| aneuploidy | 18 (28.1%) | 5 (12.2%) | NS |

| total | 64 (100%) | 41 (100%) |  |
| --- | --- | --- | --- |

1PN, single pronucleus; GW-UPD, genome-wide-uniparental diploidy; H, hour;

*: P < 0.05, **: P < 0.01; NS, No significance, The chi-square (χ2) test was used.
